# Corticosterone Upregulates Gene and Protein Expression of Catecholamine Markers in Organotypic Brainstem Cultures

**DOI:** 10.3390/ijms20122901

**Published:** 2019-06-14

**Authors:** Carla L. Busceti, Rosangela Ferese, Domenico Bucci, Larisa Ryskalin, Stefano Gambardella, Michele Madonna, Ferdinando Nicoletti, Francesco Fornai

**Affiliations:** 1 I.R.C.C.S. Neuromed, 86077 Pozzilli, Italy; carla.busceti@neuromed.it (C.L.B.); rosangela.ferese@neuromed.it (R.F.); domenico.bucci@neuromed.it (D.B.); stefano.gambardella@neuromed.it (S.G.); stabulario@neuromed.it (M.M.); nicoletti@neuromed.it (F.N.); 2 Department of Translational Research and New Technologies in Medicine and Surgery, University of Pisa, 56126 Pisa, Italy; larisa.ryskalin@unipi.it; 3 Department of Physiology and Pharmacology, University Sapienza, 00185 Roma, Italy

**Keywords:** glucocorticoids, noradrenaline, dopamine, tyrosine hydroxylase, reticular formation, dopamine transporter

## Abstract

Glucocorticoids are produced by the adrenal cortex and regulate cell metabolism in a variety of organs. This occurs either directly, by acting on specific receptors in a variety of cells, or by stimulating catecholamine expression within neighbor cells of the adrenal medulla. In this way, the whole adrenal gland may support specific metabolic requirements to cope with stressful conditions from external environment or internal organs. In addition, glucocorticoid levels may increase significantly in the presence of inappropriate secretion from adrenal cortex or may be administered at high doses to treat inflammatory disorders. In these conditions, metabolic alterations and increased blood pressure may occur, although altered sleep-waking cycle, anxiety, and mood disorders are frequent. These latter symptoms remain unexplained at the molecular level, although they overlap remarkably with disorders affecting catecholamine nuclei of the brainstem reticular formation. In fact, the present study indicates that various doses of glucocorticoids alter the expression of genes and proteins, which are specific for reticular catecholamine neurons. In detail, corticosterone administration to organotypic mouse brainstem cultures significantly increases Tyrosine hydroxylase (TH) and Dopamine transporter (DAT), while Phenylethanolamine N-methyltransferase (PNMT) is not affected. On the other hand, Dopamine Beta-Hydroxylase (DBH) increases only after very high doses of corticosterone.

## 1. Introduction

Glucocorticoids are produced from the adrenal cortex and may be administered as a drug. These compounds spread to the whole extracellular space to regulate a variety of functions. The amount of glucocorticoids present is key to regulating inflammation [1,2], cell proliferation [3], pain [4,5,6,7] and cell metabolism [8,9] in a variety of organs. In this way, glucocorticoids produce a variety of effects, which are seminal for homeostasis. The anatomical connections between the adrenal cortex and adrenal medulla, due to a specific capillary network, determine high glucocorticoids concentrations focally at the level of the adrenal medulla, where they promote catecholamine synthesis and shift noradrenaline into adrenaline cells through epigenetic effects [10,11,12,13,14]. High levels of glucocorticoids may occur during prolonged stressful conditions [15,16,17] or as a consequence of inappropriate production primarily within the adrenal cortex [18,19] or they may increase following abnormal pituitary ACTH production [20,21,22]. Similarly, exogenous administration of glucocorticoids is a common way to treat a variety of disorders, including chronic inflammatory diseases. In all these circumstances, glucocorticoids produce multiple effects, which may lead to Cushing syndrome [23]. This syndrome features altered plasma glucose level [24], abnormally high pressure [25], altered distribution of lipids in the body [26], thinning of the skin [27], and muscle atrophy, mostly in the lower limb [28]. Neurological alterations are common, featuring mood disorders [29], altered sleep-waking cycle [30], autonomic dysfunction [31], anxiety [32], and movement disorders [33]. Neuropsychiatric symptoms occurring in Cushing syndrome are still lacking a clear molecular explanation and even the brain regions being affected remain under debate. Most behavioral symptoms persist for a considerable amount of time, even when normal glucocorticoid levels are re-established back to control values. This suggests that exposure to high levels of corticosteroid produces plastic changes of neuronal phenotype, which persist over time. This is similar to the phenotypic shift which normally takes place within adrenal medulla. Thus, we wondered whether prolonged high corticosteroids levels may produce a phenotypic shift within catecholamine containing cells of central nervous system (CNS). All catecholamine nuclei are placed in a small region within the caudal part of the CNS known as the brainstem. These nuclei are highly interconnected within the brainstem reticular formation, which contains noradrenaline (NA), dopamine (DA) and adrenaline (A) cell groups. Despite these nuclei being placed in a small brain area, their axons spread to the entire CNS. This may help to understand why small nuclei, from a restricted brain region, regulate a variety of functions which are key for survival. These functions are the sleep-waking cycle, mood regulation, central control of blood pressure, and anxiety. A number of functions of these reticular nuclei overlap with those affected during exposure to high levels of glucocorticoids. In the present study we challenged the hypothesis that glucocorticoids may shift the nuclei of the reticular formation towards a catecholamine phenotype. To test this experimental issue, we selected “ad hoc” experimental settings. Since altered blood levels of corticosteroids may alter brainstem catecholamine nuclei indirectly, due to blood pressure and metabolic changes, we set up an experimental setting allowing us to establish the “pure” direct effects of glucocorticoids on brainstem reticular nuclei. Therefore, instead of an intact CNS with blood vessels and peripheral nerve connections, we used organotypic cultures of mouse brainstem to work within quite preserved short neural networks without risking the bias of non-specific systemic influences drove by blood supply or nerve afferents from the whole body. In this way we could rule out the effects of systemic glucocorticoids. Thus, a potential shift in specific gene and protein expression could be reliably attributed to a focal influence of glucocorticoids on these nuclei. This was further validated by occluding the very same effects when applying focally a glucocorticoid receptor antagonist. In order to consider the potential influence of endogenous corticosteroids in the cell growth serum (which is mandatory to sustain cell survival) we measured corticosteroid levels in the cell medium, which turned out to be way below the lowest dose administered exogenously (0.0067 µM compared with 0.1 µM, respectively). The lack of any influence in the present data of serum glucocorticoids was further demonstrated by administering the receptor antagonist, mifepristone, which did not vary the phenotype of controls, while it prevented the effects produced by exogenously administered glucocorticoids. 

## 2. Results

### 2.1. TH Increases within Organotypic Cultures Following Incubation with Corticosterone

ELISA analysis showed that the medium culture contains a negligible quantity of corticosterone (0.0067 µM) compared to those selected for the study: 0.1, 0.5, 1, and 200 µM.

**Figure 1:**
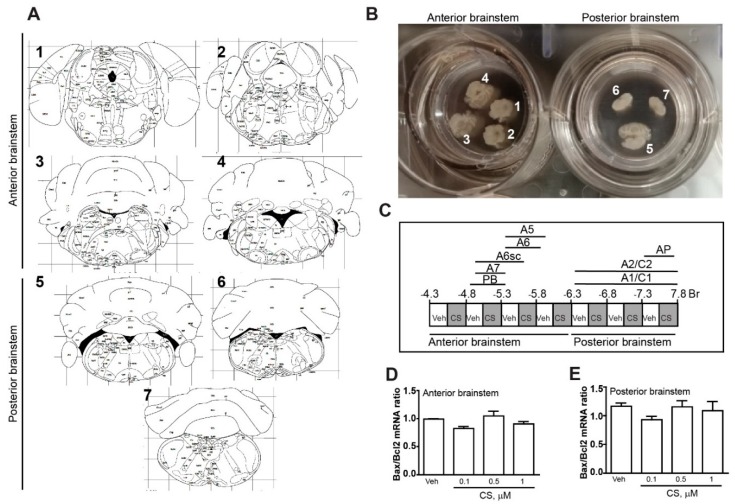
*Organotypic cultures of the mouse brainstem.* (**A**) Plates of brain atlas corresponding to the level chosen for rostral (1–4) and caudal (5–7) slices to set up organotypic cultures. (**B**) Authentic organotypic slices cultured from mouse brainstem. (**C**) Map of catecholamine nuclei of the mouse brainstem from pons to medulla oblongata from Bregma= −4.3 to Bregma= −7.8. The diagram shows the anatomical localization of each brainstem catecholamine nuclei. From each brainstem, 16 coronal sections (250 µm thickness) were sampled with a 250 µm interval. Eight sections were treated with vehicle (Veh), while 8 were administered various corticosterone (CS) doses. The diagram shows the rostro-caudal extension of the rostral (from Bregma= −4.3 to Bregma= −6.3) and the caudal part (from Bregma= −6.3 to Bregma= −7.8) of the mouse brainstem. (**D**,**E**) Real time PCR analysis of the Bax/Bcl2 mRNA ratio in organotypic cultures of the rostral or caudal part of the mouse brainstem at 24 h vehicle (Veh, ethanol 0.01%) or CS (0.1, 0.5 or 1 µM in ethanol 0.01%) incubation. The value reported for the vehicle in Figure 1D was standardized based on vehicle of Figure 1C. This means that in baseline conditions, the BAX/Bcl2 ratio is higher in the caudal compared with rostral brainstem.

These doses were selected in order to reproduce a moderate (0.1–1 µM) to massive, frankly toxicant (200 µM) corticosterone stimulation on the basis of previous studies [34,35,36,37]. In order to assess the effect of glucocorticoids on the brainstem, we exposed organotypic cultures following a subdivision of the brainstem in two blocks: the anterior part (also defined as rostral brainstem, from Bregma= −4.3 to Bregma= −6.3) and the posterior part (also defined as the caudal brainstem, from Bregma= −6.3 to Bregma= −7.8) (Figure 1A–C). 

**Figure 2:**
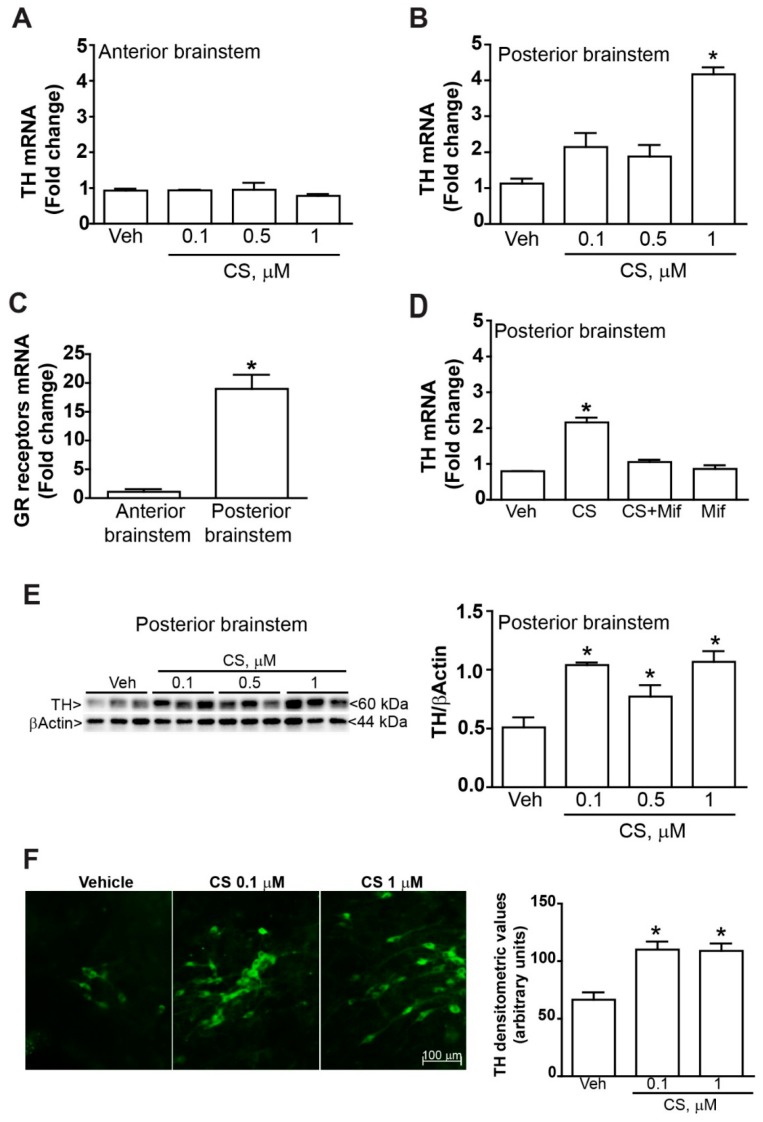
*Corticosterone increases TH expression in the mouse brainstem.* (**A**,**B**) Real time PCR analysis of TH mRNA in cultures from the rostral or caudal part from the mouse brainstem at 24 h vehicle (Veh, ethanol 0.01%) or CS (0.1, 0.5 or 1 µM in ethanol 0.01%) incubation. (**C**) Real time PCR analysis of glucocorticoid receptors (GR) mRNA. (**D**) Real time PCR analysis of TH mRNA in cultures of the caudal part at 24 h incubation with either vehicle (Veh, ethanol 0.01%) or CS (0.1 µM in ethanol 0.01%) in the presence or absence of the selective GR receptors antagonist mifepristone (Mif, 10 µM). (**E**) Western blot analysis of TH in organotypic cultures of the caudal part of the brainstem at 24 h incubation with either vehicle (Veh, ethanol 0.01%) or CS (0.1, 0.5 or 1 µM). (**F**) Immunofluorescence of TH in the A1/C1 catecholamine area of the caudal part of the brainstem at 24 h incubation with either vehicle (Veh, ethanol 0.01%) or CS (0.1, or 1 µM). Densitometry of immunofluorescence was expressed in arbitrary units. All values are expressed as the means±SEM. * *p* < 0.05 compared with vehicle.

Corticosterone concentrations ranging from 0.1 to 1 µM did not induce apoptosis, as shown by unchanged value of Bax/Bcl2 mRNA ratio (Figure 1D,E). However, real time PCR analysis showed a marked upregulation of TH mRNA level in the caudal part which was absent in the rostral part (Figure 2A), while the effect was significant in the caudal part (Figure 2B). Such a rostro-caudal difference is consistent with a remarkable amount of glucocorticoid receptors mRNA levels in the caudal compared with negligible amount in the rostral part of the mouse brainstem (Figure 2C). The glucocorticoid receptor–dependency of such an effect was confirmed by administering the selective glucocorticoid receptor antagonist mifepristone (10 µM), which suppresses the increase of TH mRNA levels in the caudal part of the brainstem (Figure 2D). Remarkably, mifepristone did not modify TH mRNA levels measured in controls, which indicates a lack of effective stimulation of glucocorticoid receptors potentially induced by glucocorticoids detectable in trace amounts within horse serum or even in the medium culture. The increase in TH mRNA levels was consistent with glucocorticoid-induced increase in the TH protein as roughly measured by Western blot analysis (Figure 2E), in very same part of the brainstem for the very same corticosterone doses (0.1, 0.5 or 1 µM, at 24 h). Consistently, immunohistochemistry provided evidence for an increase in TH-immune-staining affecting the caudal part of the brainstem, mostly at the level of A1/C1 nuclei (Figure 2F).

**Figure 3:**
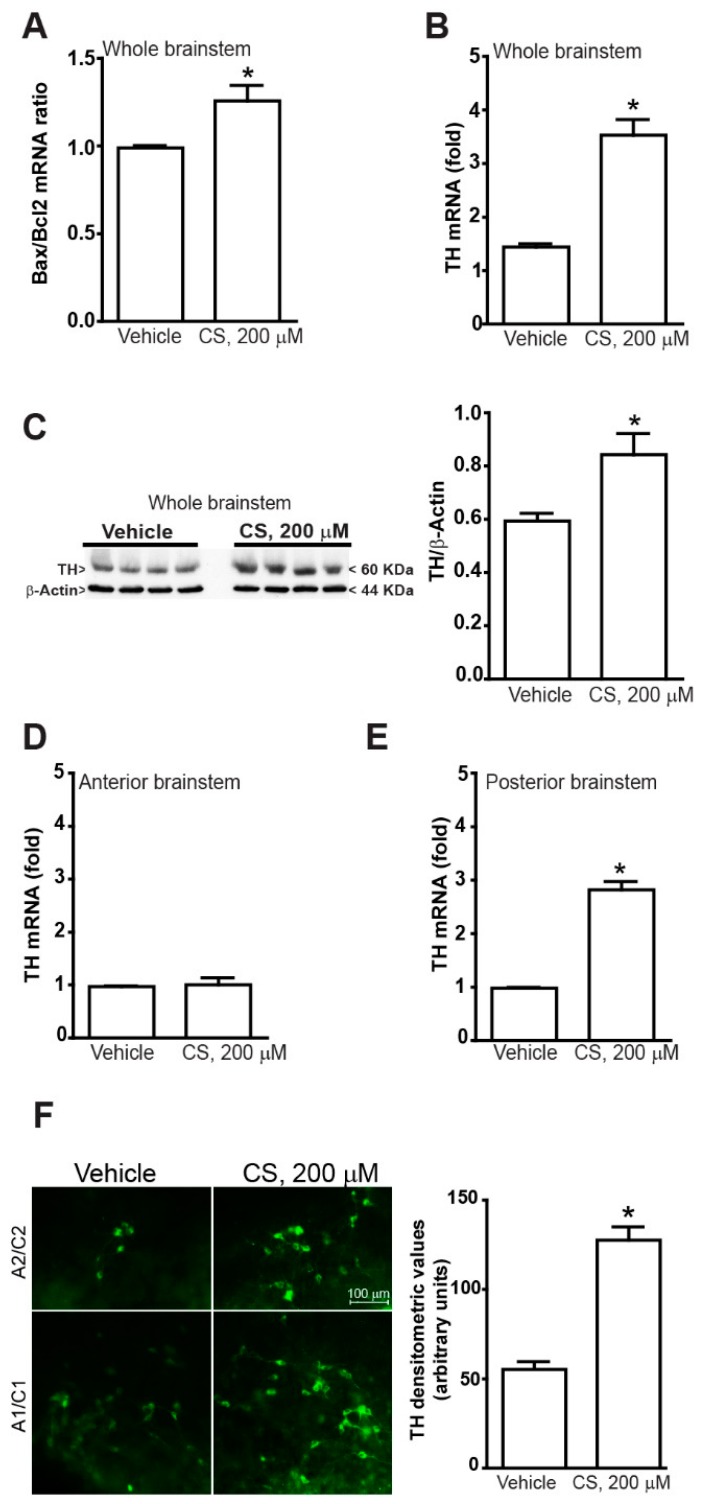
A high dose of corticosterone increases TH expression in the mouse brainstem. (**A**) Real time PCR analysis of the Bax/Bcl2 mRNA ratio in the whole mouse brainstem at 24 h incubation with either vehicle (Veh, ethanol 0.66%) or CS (200 µM in ethanol 0.66%). (**B**) Real time PCR analysis of TH mRNA level and (**C**) western blot analysis of TH protein within the whole mouse brainstem at 24 h incubation with either vehicle (Veh, ethanol 0.66%) or CS (200 µM in ethanol 0.66%). (**D**,**E**) Real time PCR analysis of TH mRNA within the rostral or caudal and part of the brainstem at 24 h incubation with either vehicle (Veh, ethanol 0.66%) or CS (200 µM in ethanol 0.66%). (**F**) Immunofluorescence of TH in the A1/C1 and A2/C2 catecholamine areas of the caudal brainstem at 24 h incubation with either vehicle (Veh, ethanol 0.66%) or CS (200 µM in ethanol 0.66%). Densitometry of immunofluorescence is expressed in arbitrary units. All values are expressed as the means ± SEM. * *p* < 0.05 compared with the vehicle.

In a separate set of experiments, we assessed the effect of a neurotoxic concentration of corticosterone on the catecholamine system of the mouse brainstem. To this aim, organotypic cultures from the mouse brainstem were treated for 24 h with 200 µM of corticosterone [38,39,40]. The neurotoxic effect was detected as a significant increase of the Bax/Bcl2 mRNA ratio in cultures of whole mouse brainstem at 24 h of incubation with the highest dose of corticosterone (Figure 3A). Despite a pro-apoptotic evidence, real time PCR and western blot analyses following the highest dose of corticosterone indicate a robust increase in the TH mRNA (Figure 3B) and protein (Figure 3C) when measured from the whole mouse brainstem. However, no effect was observed in the rostral part (Figure 3D), thus the increase of TH mRNA was selectively due to a remarkable effect within the caudal part (Figure 3E). This recapitulates what observed for low doses of corticosterone. This was confirmed by the increased density of TH immune-staining in A1/C1 and A2/C2 regions of the caudal brainstem (Figure 3F).

### 2.2. Low Doses of Corticosterone Increase DAT mRNA and Protein without Altering PNMT and DBH 

**Figure 4:**
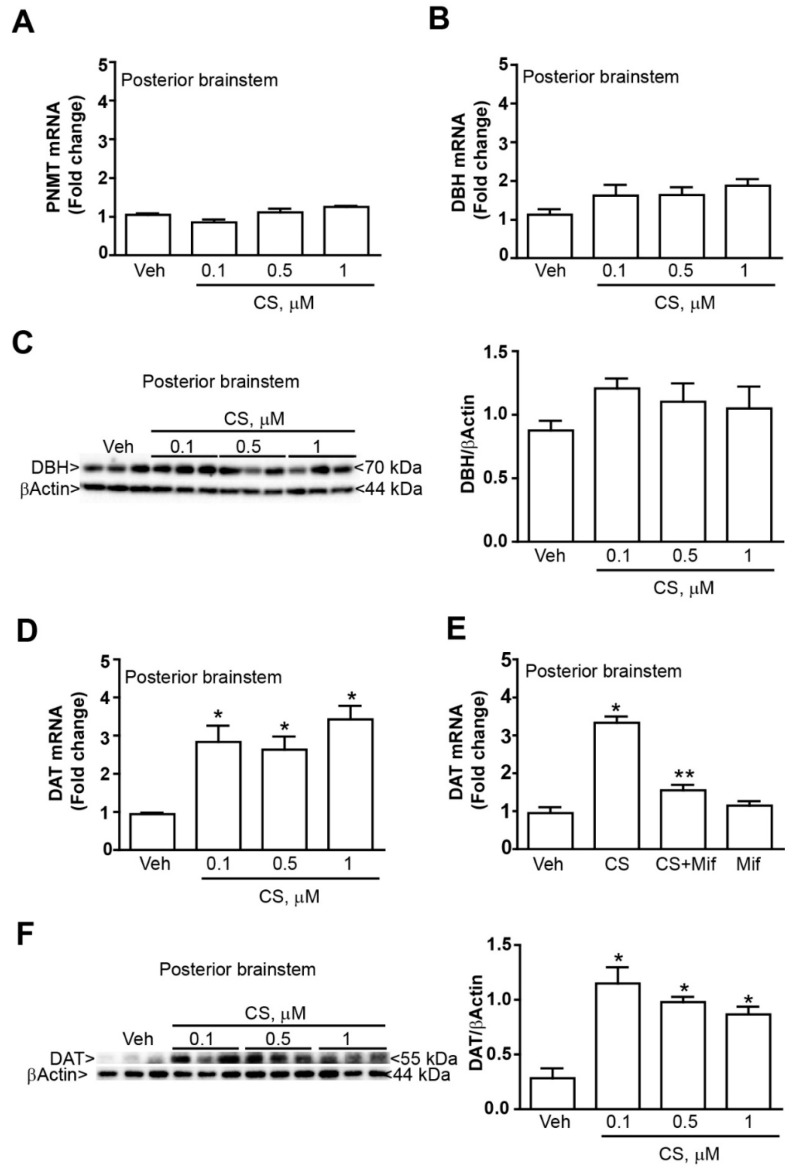
*Corticosterone increases DAT expression without modifying DBH and PNMT expression in the mouse brainstem*. Real time PCR of (**A**) the adrenergic marker PNMT and (**B**) the noradrenergic marker DBH within the caudal part of the brainstem at 24 h incubation with either vehicle (Veh, ethanol 0.01%) or corticosterone (CS, 0.1, 0.5 or 1 µM). (**C**) Western blot of DBH within the caudal part of the brainstem at 24 h incubation with either vehicle (Veh, ethanol 0.01%) or corticosterone (CS, 0.1, 0.5 or 1 µM). (**D**) Real time PCR of the dopaminergic marker (DAT) within the caudal part of the brainstem at 24 h incubation with either vehicle (Veh, ethanol 0.01%) or corticosterone (CS, 0.1, 0.5 or 1 µM). (**E**) Real time PCR of the DAT within the caudal part of the brainstem at 24 h incubation with either vehicle or CS (0.1 µM), in the presence or absence of the selective glucocorticoid receptors antagonist mifepristone (Mif, 10 µM). (**F**) Western blot of DAT within the caudal part of the brainstem at 24 h incubation with either vehicle (Veh, ethanol 0.01%) or corticosterone (CS, 0.1, 0.5 or 1 µM). The lines of the βActin are the same showed in the Figure 2E as the western blot for DAT was performed by incubating with the anti-DAT primary antibody the very same membrane used for TH immunoblotting. All values are expressed as the means±SEM. * *p* < 0.05 compared with the vehicle. ** *p* < 0.05 compared with the vehicle and CS.

We examined whether TH increased expression was associated with changes in the expression of the noradrenergic marker DBH and/or the adrenergic marker PNMT. Our data did not show any differences in the expression of DBH and PNMT mRNA levels in organotypic cultures of the caudal part or the whole brainstem in response to treatment with low corticosterone doses (0.1, 0.5 or 1 µM) (Figure 4A,B). Similarly, Western blot analysis did not show any difference in DBH expression level in the caudal part of the brainstem in response to low concentrations of corticosterone (0.1, 0.5 or 1 µM) (Figure 4C). On the other hand, mRNA expression level of the dopaminergic marker DAT was significantly increased in cultures from the caudal part of the brainstem (Figure 4D). This effect depends on the stimulation of corticosterone receptor since it was occluded by the receptor antagonist mifepristone (Figure 4E). It is remarkable that the very same dose of mifepristone did not modify the DAT mRNA expression level in vehicle-treated cultures, thus ruling out an effective role for the negligible amount of corticosteroid we measured in the culture medium.

Similarly to mRNA levels, Western blotting of the DAT indicates an increased protein level in organotypic cultures from the caudal part of the brainstem (Figure 4F).

**Figure 5:**
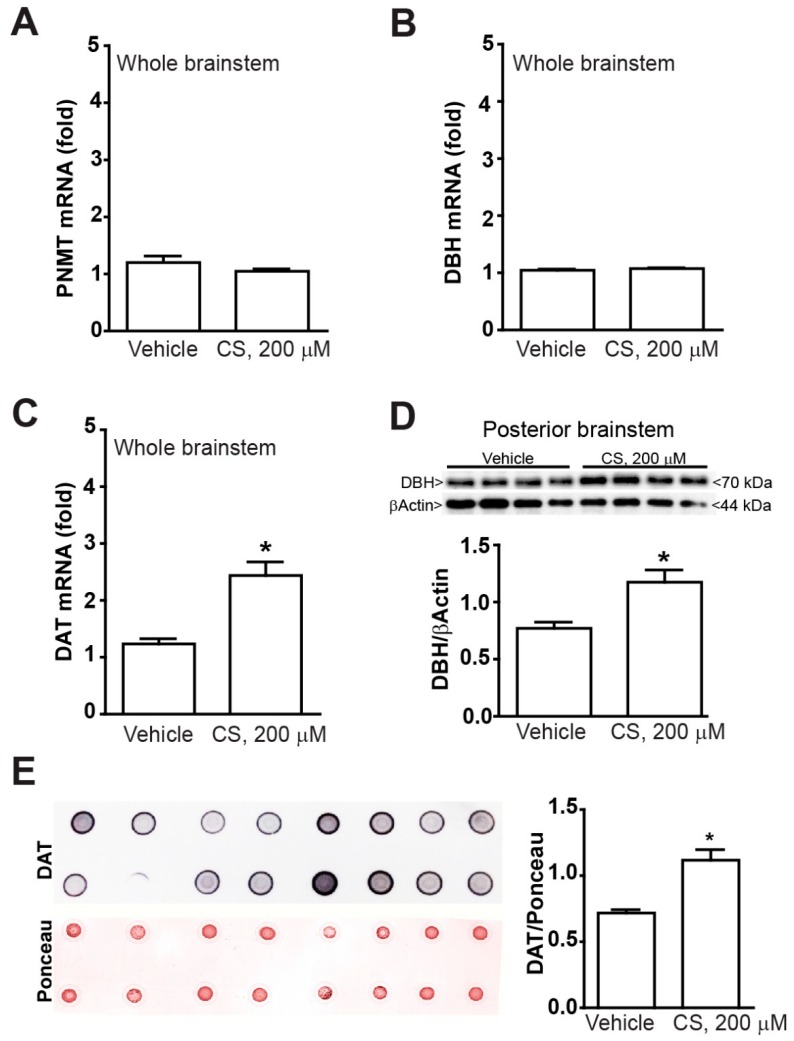
*The highest dose of corticosterone increases DBH expression without modifying PNMT expression in the mouse brainstem.* Real time PCR of (**A**) the adrenergic marker PNMT, (**B**) the noradrenergic marker DBH and (**C**) the dopaminergic marker (DAT) in the whole mouse brainstem at 24 h incubation with either vehicle (Veh, ethanol 0.66%) or CS (200 µM in ethanol 0.66%). (**D**) Western blot of DBH within the caudal part of the brainstem at 24 h incubation with either vehicle (Veh, ethanol 0.66%) or CS (200 µM in ethanol 0.66%). (**E**) Dot blots of DAT in the whole mouse brainstem at 24 h incubation with either vehicle (Veh, ethanol 0.66%) or CS (200 µM in ethanol 0.66%) support data shown at Western blotting. Values are expressed as the means±SEM. * *p* <0.05 compared with the vehicle.

When the highest doses of corticosterone was administered, PNMT and DBH mRNA levels were still similar to the vehicle when measured in the whole brainstem (Figure 5A,B). In contrast, the highest dose of corticosterone increased mRNA DAT levels even when measured in the whole brainstem (Figure 5C). Unexpectedly, when the DBH protein was immunoblotted in the caudal brainstem, the highest dose of corticosterone produced a significant increase (Figure 5D), which was not documented following the low doses. This suggests that despite the DA phenotype being triggered by low doses of corticosterone, a noradrenergic phenotype may be induced by corticosterone on the mouse brainstem during extreme corticosterone stimulation. 

Incidentally, when the DAT levels were tested with Ponceau-stained dot blotting the highest dose of corticosterone replicated the increase in DAT protein documented in the brainstem for the whole range of low doses at western blotting. 

## 3. Discussion

In the present manuscript, an increased expression of specific catecholamine-related genes and proteins within mouse caudal brainstem reticular nuclei is documented. These effects occur differently following different doses of corticosterone, ranging from the effects occurring during stressful conditions, or during Cushing syndrome, up to a high toxic concentration.

These data were obtained using organotypic cultures from the mouse brainstem. The cultures were obtained by dissecting thick slices sampled along the whole rostro-caudal extent of the pons and medulla oblongata, ruling out the midbrain.

This experimental setting allowed us to assess the pure effect of corticosterone exposure on the catecholamine system within brainstem local networks, while ruling out the potential bias due to systemic effects induced by whole body corticosterone administration.

These organotypic cultures allow visualizing neuronal aggregates in their coronal extent with a fair preservation of local circuitries. Of course, the effects of distant neural connections are lost in these experimental conditions, although the authentic effects of corticosterone on catecholamine markers can be inferred more reliably. At caudal brainstem level, a marked increase in the catecholamine enzyme TH is documented. This is a key finding since TH is the rate-limiting step in catecholamine biosynthesis. The increase of TH documented here consisted both in mRNA and protein level. The tissue immunofluorescence confirmed increased density of TH immune-staining at the level of A1/C1 area in the caudal brainstem. Remarkably, the *Th* gene owns highly conserved glucocorticoid-responsive elements [41,42]. In the present study, the susceptibility of the caudal compared with rostral brainstem to corticosterone-induced TH expression was found to be consistently related to the impressive expression of glucocorticoid receptors in the caudal brainstem compared with the rostral brainstem. The site-specific regulation of TH expression according to a regional gradient of glucocorticoids receptors may underlie phenotype development of catecholamine neurons under glucocorticoids stimulation, which appear at the birthdate in rodent pups [43].

High density of glucocorticoid receptors also occurs within mesencephalic DA nuclei where, based on their nuclear translocation they are supposed to alter cell phenotype to influence the meso-limbic and meso-striatal DA system [44]. Within this context, it is noteworthy that the amount of meso-striatal DA axons is increased following administration of prednisolone in rodents following damage with the DA neurotoxin 6-hydroxydopamine (6-OHDA) [45]. Remarkably, DA uptake was already reported to be increased as a consequence of increased DAT expression in mice following restraint stress [46]. Nonetheless, so far no study was able to assess an epigenetic mechanism underlying these effects. Further studies analyzing the innumerous connections among the cascade which regulate gene expression will eventually elucidate the molecular mechanism underlying the glucocorticoid regulation of the DAT gene. Such an effort is expected to produce some results during future years since so far, no evidence in available genomic database is present to point to a significant molecular hypothesis. A similar conundrum exists for the *Dbh* gene which in our extreme experimental conditions (the highest dose of corticosterone) was increased as well. 

In line with previous studies in the midbrain, the catecholamine nuclei being investigated here also possess glucocorticoid receptors [47,48,49,50,51]. 

This is in line with data obtained in the present study showing an increase in TH mRNA and protein levels in the brainstem, which correspond to a higher TH immunoreactivity as shown in representative figures of A1/C1 as well as A2/C2. These data suggest that glucocorticoid administration produces a phenotypic shift towards a catecholamine phenotype of hind brainstem reticular neurons. The present data do not contradict the finding of Makino et al. [52] and Zhang et al. [53] who published that stress was also inducing TH expression in a glucocorticoid-independent manner. 

In fact, the experimental setting provided by organotypic cultures allowed us to dissect only the direct effects of glucocorticoids on these nuclei independently of systemic effects (increased blood pressure, altered blood glucose and others) which may be produced by systemic glucocorticoids levels, which may in fact increase TH expression in a glucocorticoid-independent manner.

A specific point of the manuscript consists in the measurement of glucocorticoid effects in the caudal brainstem catecholamine nuclei. In order to explain the different response to in vitro treatment with corticosterone between the anterior and the posterior portion of the mouse brainstem, we performed a real time PCR quantification of glucocorticoid receptor mRNA. This study allowed for demonstrating higher level of receptor expression in the posterior compared with the anterior mouse brainstem. This is in line with the data provided here and it confirms the high expression of glucocorticoid receptors in the catecholamine cell groups of the posterior brainstem described by Harfstrand et al. [48].

Remarkably, there may be opposite changes in TH expression depending on which catecholamine nucleus in the reticular formation is considered. Thus, LC which possess a low amount of glucocorticoid receptor appear to undergo a reduced TH expression under the effects of glucocorticoids [49], which confirms a reduced number of glucocorticoid receptors we measured here at rostral level as well as the lack of response of these nuclei to glucocorticoids. In contrast, the nucleus of the solitary tract (which roughly correspond to ala cinerea and the A2/C2 area in its catecholamine containing cells) was reported to increase TH content following exposure to glucocorticoids [54]. Again, this confirms the impressive amount of glucocorticoid receptor at caudal level of the brainstem measured here (Figure 2C). Apart from glucocorticoids modulation of TH expression, to our knowledge, the present study was the first to investigate the expression of mRNA and protein levels of DAT, which is specific DA marker. Despite no alterations were observed neither for PNMT nor for DBH at low doses, glucocorticoids induce an increase in mRNA and protein levels for DAT in the caudal brainstem reticular formation. Remarkably, glucocorticoids may modulate DAT expression in the lower brainstem, which is critical for a variety of functions. So far, increased DAT expression was only investigated in the midbrain by Virdee et al. [55]. These authors demonstrated that antenatal glucocorticoid exposure increases in the adulthood the number of midbrain DA neurons concomitantly with striatal DA fibers. just like what was previously shown in the adult substantia nigra and VTA [56,57]. Consistently, Niwa et al. [58] demonstrated that glucocorticoids mediate the stress-induced increase in the expression of TH within midbrain DA neurons in the adolescent brain. Altogether, these findings demonstrated that midbrain DA neurons are sensitive to glucocorticoids, which increase their catecholamine phenotype. The findings provided here extend these effects to catecholamine neurons of the caudal brainstem showing that a potential phenotypic shift takes place within reticular nuclei due to an increase in DA markers. This is expected to play a key role in the effects produced by the caudal catecholamine cell groups such as blood pressure, breathing, autonomic functions, alertness and anxiety. Remarkably, these correspond to some items, which vary during the Cushing syndrome.

## 4. Materials and Methods 

### 4.1. Organotypic Brainstem Cultures

For organotypic brainstem, cultures were used postnatal day 18 C57Bl6/J derived from breading in house C57Bl6/J mice (Charles River, Calco, LC, Italy). The animals were rapidly sacrificed, brains dissected and coronally cut with the vibratome Leica VT1200S (Leica Biosystems, Buccinasco, MI, Italy). Slices of 250 µm of thickness were cut along the whole rostro-caudal extension of the pons and medulla oblongata (from Bregma= −4.3 to Bregma= −7.8) without including the midbrain (Figure 1A). We obtained 16 sections/brainstem for an extension of 4 mm. All sections were collected in sterile artificial cerebrospinal fluid (ACSF, 1 mM calcium chloride, 10 mM D-glucose, 4 mM potassium chloride, 5 mM magnesium chloride, 26 mM sodium bicarbonate, 246 mM sucrose) and placed onto a sterile 0.4 µm pore membrane (Merck Millipore, Billerica, MA, USA, Cat# PICM03050, lot n°: R6DA39745) within a 6-well plate. Sections were cultured in 6-well plates at 37 °C and 5% CO2 with 1 mL/well of the following culture medium: 50% MEM/HEPES, 25% Heat Inactivated Horse Serum, 25% Hank’s solution, 2 mM NaHCO3, 6.5 mg/mL glucose, 2 mM glutamine, 100 IU/mL penicillin and 100 mg streptomycin, pH 7.2. Sections were incubated for five days and the medium was changed every two days. All animal experiments were approved by Italian Ministry of Health (N° 1065/2016-PR, 7 November 2016).

### 4.2. Corticosterone ELISA

The corticosterone levels in the culture medium was measured with a corticosterone enzyme-linked immunosorbent assay kit (ELISA; Abcam, Minneapolis, MN, code: ab108821; lot number: GR321740-9), according to the manufacturer’s instructions.

### 4.3. Experimental Planning

For cultures of whole mouse brainstem, from each brainstem we obtained 16 sections (250 µm thickness) for an extension of 4 mm (from Bregma= −4.3 to Bregma= −7.8). Of these, 8 (sampled every 500 µm) were treated with vehicle (0.01% ethanol as control for CS 0.1, 0.5 or 1 µM and 0.66% ethanol as control for CS 200 µM) and 8 (sampled every 500 µm) with different concentrations of corticosterone (Sigma Aldrich, Milan, Italy, Cat# C2505; lot n°: SLBJ5337V). For cultures of the anterior portion of the mouse brainstem, sections sampled from Bregma -4.3 to Bregma -6.3 were used. For cultures of the posterior portion of the mouse brainstem, sections sampled from Bregma -6.3 to Bregma −7.8 (Figure 1B) were used. The concentration of corticosterone used for the experiments were selected based on previous studies in order to expose the cultures to stress-like/Cushing-like concentrations (CS: 0.1; 0.5 or 1 µM; [34,35,36,37]) or to a neurotoxic paradigm (CS: 200 µM; [38,39,40]). 

The corticosterone was dissolved in 0.01% ethanol for CS 0.1, 0.5 and 1 µM or in 0.66% ethanol for CS 200 µM. The groups of control were incubated with vehicle (0.01% ethanol or 0.66% ethanol).

Following 24 h of incubation, the organotypic brainstem cultures were pooled (from the whole brainstem: from Bregma= −4.3 to Bregma= −7.8; or from the anterior brainstem: from Bregma= −4.3 to Bregma= −6.3 and the posterior brainstem from Bregma= −6.3 to Bregma= −7.8) and used for Real-Time PCR analyses of Bax, Bcl2, *Th*, *Dbh*, *Pnmt*, *Dat* and *GR* (*Glucocorticoid receptor*) mRNA levels. Separate cultures were subjected to the same experimental condition and used for western blot analysis of TH, dot blot analysis of DBH and DAT or immunohistochemical analysis of TH.

In additional set of experiments, organotypic cultures of the posterior portion of the mouse brainstem were treated for 24 h with CS 0.1 µM in the presence of absence of the selective glucocorticoid receptor antagonist mifepristone (10 µM, Sigma Aldrich, Cat# M8046-100MG; lot n°: WXBC5203V). At the end of incubation, the slices were pooled and used for Real-Time PCR analyses of *Th* and *Dat* mRNA levels. 

### 4.4. Western Blot

Tissues were homogenized at 4°C in ice-cold lysis buffer with phosphatase and protease inhibitor. Twenty-five µg of proteins were incubated for 1 h with a mouse monoclonal anti-TH (1:10000, Sigma Aldrich, Cat# T1299 RRID:AB_477560; lot n°: 015M4759V) or a mouse monoclonal anti β-Actin (1:50000, Sigma Aldrich, Cat# A5441 RRID:AB_476744, lot n°: 028K4826) antibodies and then with secondary peroxidase coupled anti-mouse antibody (1:3000; Merck Millipore, Calbiochem^®^, Cat# 401215-2ML RRID:AB_437766; lot n°: 2782376). Immunostaining was revealed by enhanced chemiluminescence luminosity (GE Healthcare, Milan, Italy). Densitometric analysis was performed with ImageJ software.

### 4.5. Dot Blot

For dot blot analyses 1.2 µg of proteins were spotted onto the nitrocellulose membranes. The membranes were blocked for 2 h with 5% non-fat dry milk and then incubated overnight with primary monoclonal rat anti-DAT (1:1000, Merck Millipore, Cat# MAB369 RRID:AB_2190413; lot n°: 2664466) or monoclonal mouse anti-DBH (1:1000, Merck Millipore, Cat# MAB308 RRID:AB_2245740; lot n°: 2688638) antibodies. Filters were washed 3 times with TTBS buffer and then incubated for 1 h with secondary peroxidase-coupled anti-rat (1:3000, Merck Millipore, Calbiochem^®^, Cat# 401416 RRID:AB_437801; lot n°: D00170203) or anti-mouse (1:3000, Merck Millipore, Calbiochem^®^, Cat# 401215-2ML RRID:AB_437766; lot n°: 2782376) antibodies. Immunostaining was revealed by enhanced chemiluminescence luminosity (GE Healthcare). For the normalization process, membranes were stained with Ponceau S (Sigma Aldrich, Cat# P3504, lot n°: MKBF2200V, 1 mg/mL in Acetic acid/H2O 1/20) for 10 s. Densitometric analysis was performed with ImageJ software.

### 4.6. Real Time PCR Analysis

Total RNA was isolated using Trizol Reagent (Thermo Fisher Scientific, Invitrogen™, Waltham, MA, USA, Cat# 10296010, lot n°: 69083302) according to the manufacturer’s instructions. The concentration and purity of RNA samples were determined using Nanodrop 2000 (Thermo Fisher Scientific, Life Technologies). Total RNA (100 ng) was reverse transcribed (RT) with SuperScript^®^ VILOTM (Thermo Fisher Scientific, Invitrogen™, Cat# 100011931, lot n°: 1807301) with Oligo dT primers. 

Amplification and detection were performed on a CFX Connect™ Real Time System (Bio-Rad, Hercules, CA, USA). PCR mix including 10 μL SYBR Green PCR Master (Applied Biosystems, Foster City, CA, USA, Cat# 4367659, lot n°: 1610538), 0,5 μM of each primer and 1 μL of RT reaction mix, was amplified as follows: 95°C for 10 min followed by 40 cycles of 95°C for 1 min and 30 s, 54°C for 1 min. The following primers have been designed using GenBank (http://www.ncbi.nlm.nih.gov/): *Bax* (NM_007527): 5′-aagctgagcgagtgtctc- 3′, 5′-agttgaagttgccatcagc-3′; *Bcl2* (NM_009741): 5′-ggtggtggaggaactctt-3′, 5′-ggctgagcagggtcttca-3′; *Th* (NM_009377): 5′-acctggagtactttgtgcg-3′, 5′-ctttgtgtattccacgtgtg-3′; *Dbh* (NM_138942): 5′-agccaagactaccagctgc-3′, 5′-gtagctcagtgatatagca-3′; *Pnmt* (NM_008890): 5′-acatcaccatgacagactt-3′, 5′-ctggaagctagtaagatct-3′; *Dat* (NM_010020): 5′-cacagaggactcgagatc-3′, 5′-atgcaggcctgaggtgtt-3′, *GR* 5′-aatgagaccagatgtgagttc-3′, 5′-tagcggcatgctggaca-3′ and *Beta-Globin* (NM_008219): 5′-ctaaggtgaaggctcatg- 3′; 5′-gataggcagcctgcact-3′.

Predesigned TaqMan Gene Expression assays for *Th* was obtained from Applied Biosystems. *Gapdh* was used as the endogenous control to confirm the correct work to SYBR assay.

Positive controls (DNA), negative control (distilled water), and RT-negative controls (total RNA sample) were included in each run.

The relative quantification was calculated using comparative Ct method (also known as the ΔΔ*C*T method) [59,60] and *Beta-Globin* and *Gapdh* were selected as internal references. Ct values correspond to mean values of each PCR performed in triplicate. Gene expression was confirmed in two independent experiments.

### 4.7. Immunohistochemistry

Following incubation with corticosterone (200 µM, 24 h), cultures were fixed in 2.5% PFA/4% sucrose for 1.5 h and incubated overnight with a mouse monoclonal anti-TH (1:100; Sigma Aldrich, Cat# T1299 RRID:AB_477560; lot n°: 015M4759V) and then for 1 h with a secondary fluorescein anti-mouse antibody (1:50; Vector Laboratories, Burlingame, CA, Cat# BA-1000 RRID:AB_2313606; lot n°: ZA0324).

### 4.8. Statistical Analysis

All analyses were performed using the Student’s t test (Figure 2C, Figure 3 and Figure 5) or One-way Anova + Fisher post hoc (Figure 1, Figure 2A,B,D–F and Figure 4). Differences at *p* < 0.05 were considered statistically significant.

## Abbreviations

6-OHDA	6-hydroxydopamine
A	Adrenaline
CNS	Central nervous system
CS	Corticosterone
DA	Dopamine
DAT	Dopamine transporter
DBH	Beta-Hydroxylase
GR	Glucocorticoid receptors
LC	Locus coeruleus
NA	Noradrenaline
PNMT	Phenylethanolamine N-methyltransferase
TH	Tyrosine hydroxylase
VTA	Ventral tegmental area
